# Asymptotic Expansion Method with Respect to Small Parameter for Ternary Diffusion Models

**DOI:** 10.1007/s12539-017-0228-5

**Published:** 2017-04-12

**Authors:** Marek Danielewski, Henryk Leszczyński, Anna Szafrańska

**Affiliations:** 1 Faculty of Materials Science and Ceramics, AGH University of Science and Technology, 30 Mickiewicza Av., 30-059 Krakow, Poland; 2 Institute of Mathematics, University of Gdańsk, Wit Stwosz Street 57, 80-952 Gdańsk, Poland; 3 Department of Differential Equations and Mathematics Applications, Gdańsk University of Technology, G. Narutowicz Street 11/12, 80-233 Gdańsk, Poland

**Keywords:** Diffusion, Mass conservation, Difference scheme, Asymptotic series expansion, Small parameter, 35K57, 35C20, 65M06

## Abstract

Ternary diffusion models lead to strongly coupled systems of PDEs. We choose the smallest diffusion coefficient as a small parameter in a power series expansion whose components fulfill relatively simple equations. Although this series is divergent, one can use its finite sums to derive feasible numerical approximations, e.g. finite difference methods (FDMs).

## Introduction

The subject of divergent series dates back to the distant past and is linked with the names of outstanding mathematicians such as Euler, Poincaré, Borel, Padé, and Birkhoff. For more details and additional references we refer to articles by Tucciarone [1] and Ferraro [2]. The idea of the asymptotic series comes from Poincaré, who introduced the definition of an asymptotic expansion. Nowadays, the importance of methods which are based on asymptotic expansions of solutions in small or large parameters series has grown considerably in many branches of physical, chemical, biological and engineering sciences. We mention several examples among a wide range of applications, and the list is not exhaustive. The fundamental problem of reconstruction of physical observables, based on divergent power series expansions, has been considered in many areas of quantum physics. Dyson already showed in his pioneering work [3] that expansions in quantum electro-dynamics diverge factorially. After that, it was found that divergent perturbation expansions of quantum physics are very common [4, 5]. This observation led to a substantial amount of research works in various areas of quantum physics, for example in quantum field theory [6–8] or in quantum mechanics [9, 10]. The asymptotic analysis of singularly perturbed problems is performed in the monograph [11], based on expansions of analytical solutions in the power series with respect to a small parameter. In the light of this approach there are discussed models of populations, epidemiological problems, classical models of fluid dynamics and many others. The paper [12] deals with the method of asymptotic expansions of solutions to a singularly perturbed system of integro-differential equations in epidemiology with two small parameters. Asymptotic solutions are constructed by the Tikhonov-Vasil’eva method of boundary functions.

This work concerns a system of diffusion equations. When the number of components exceeds two, the term ‘interdiffusion’ or ‘cross-diffusion’ is often used, see [13] in material sciences and [14] in life sciences. The drift in each of the PDEs depends on the gradient of all other variables, so these models exhibit the phenomenon of cross-diffusion. This makes the PDE system strongly coupled. In addition, the coefficients depend on the unknown functions and the parabolicity of this system is conditional, namely it can be proved only if solutions remain in an admissible set. The invariance of this set is connected to mass conservation laws. Any violation of this property leads to serious analytical and computational problems. We overcome these problems by constructing conservative numerical schemes, i.e. preserving a discrete version of mass. In spite of mathematical and numerical difficulties, three dimensional interdiffusion problems are still of interest. Interdiffusion becomes an important topic in electrochemistry and biochemistry, e.g. molecular channels (nano-channels) [15].

### Preliminaries

Let $\Omega$ be a bounded domain in $\mathbb {R}^n$ with a smooth boundary $\partial \Omega$. The closure of $\Omega$ is denoted by $\bar{\Omega }$. In this manuscript, we investigate the system of diffusion equations

1 $$\begin{aligned} \left\{ \begin{array}{ll} \frac{\partial u}{\partial t} =&{} \nabla \cdot \left( D_1 \nabla u - u\ \nu ^D\right) \\ \frac{\partial v}{\partial t} =&{} \nabla \cdot \left( D_2 \nabla v - v\ \nu ^D\right) \\ \frac{\partial w}{\partial t} =&{} \nabla \cdot \left( D_3 \nabla w - w\ \nu ^D\right) \\ \end{array} \right. \end{aligned}$$

on $[0,T]\times \Omega$, where $D_1, D_2, D_3>0$ and $\nu ^D$ is the drift velocity given by

$$\begin{aligned} \nu ^D = \nabla (D_1 u + D_2 v +D_3 w). \end{aligned}$$

The system (1) is considered with the initial conditions

2 $$\begin{aligned} u(0,x) = u_0(x),\quad v(0,x) = v_0(x),\quad w(0,x) = w_0(x)\ \text {for}\ x\in \bar{\Omega }, \end{aligned}$$

and the Neumann boundary conditions

3 $$\begin{aligned} \frac{\partial u}{\partial \mathbf {n}} =\frac{\partial v}{\partial \mathbf {n}} = \frac{\partial w}{\partial \mathbf {n}} = 0\;\; \text {on}\;\;\partial {\Omega }. \end{aligned}$$

where $\mathbf {n}$ denotes the outward normal vector to the boundary $\partial \Omega$.

Our analysis of the strongly coupled system (1) starts from the simplest case $w\equiv 0$. Consider two chemical substances and their normalized densities $u,v\ge 0$, $u+v\equiv 1$ described by the system of differential equations

4 $$\begin{aligned} \left\{ \begin{array}{ll} \frac{\partial u}{\partial t} =&{} \nabla \cdot \Big ( D_1 \nabla u - u\ \nabla (D_1 u+D_2 v)\Big ) \\ \frac{\partial v}{\partial t} =&{} \nabla \cdot \Big ( D_2 \nabla v - v\ \nabla (D_1 u+D_2 v)\Big ) \\ \end{array} \right. \end{aligned}$$

It is clear that the assumptions on initial functions $u_0, v_0\ge 0$ and $u_0+v_0\equiv 1$ imply $u,\; v\ge 0$, $u+v\equiv 1$. Indeed, consider new dependent variables *p*, *q* defined as linear combinations of the functions *u*, *v*:

5 $$\begin{aligned} p=u+v,\;\;\; q=D_1 u+D_2v. \end{aligned}$$

Then we obtain the following system

6 $$\begin{aligned} \left\{ \begin{array}{ll} \frac{\partial p}{\partial t} =&{} \nabla \cdot \Big ( \nabla q - p\ \nabla q\Big ) \\ \frac{\partial q}{\partial t} =&{} \nabla \cdot \Big ( -D_1 D_2 \nabla p+(D_1+D_2)\nabla q - q\ \nabla q\Big ) \end{array} \right. \end{aligned}$$

Since $u_0+v_0\equiv 1$ it is seen that $p\equiv 1$ solves the first equation of system (6). Based on the relation $v\equiv 1-u$ we obtain from (4) partial differential equation

$$\begin{aligned} \frac{\partial u}{\partial t} = \nabla \cdot \Big [ (D_1(1-u) + D_2u))\nabla u\Big ]. \end{aligned}$$

Therefore, the implication $0\le u_0\le 1 \; \Rightarrow \; 0\le u\le 1$ follows easily from the maximum principle.

There are various numerical techniques for approximations of solutions of the ternary diffusion models (1)–(3). In particular, we refer to the papers [16, 17]. The authors of [16] analyze the method of lines for diffusion equations. The paper [17] deals with iterative methods for this problem. The authors of both papers study stability and convergence of the constructed method in $L^2$ and in the Sobolev space $W^{1,\infty }$. In this paper, as a tool for approximation of solutions to the initial boundary value problems for systems of equations obtained by an asymptotic expansion, we use a finite-difference methodology. In the construction of difference schemes we apply general ideas presented in [18, 19].

## Small Parameter Method

Suppose that initial functions $u_0,v_0,w_0\in C^2(\bar{\Omega },\mathbb {R})$ in (2) satisfy the relation $u_0+v_0+w_0=1$ for $x\in \bar{\Omega }$. In the sequel we assume that the diffusion coefficients $D_1,D_2,D_3$ in the system (1) satisfy the relation $D_1>D_2>D_3>0$. If *u*, *v*, *w* satisfy (1)–(3), then $u+v+w\equiv 1$ and the system (1) reduces to the system of two equations

$$\begin{aligned} \left\{ \begin{array}{ll} \frac{\partial u}{\partial t} =&{} \nabla \cdot \left( D_1 \nabla u - u\Big [(D_1-D_3)\nabla u+ (D_2-D_3)\nabla v\Big ] \right) \\ \frac{\partial v}{\partial t} =&{} \nabla \cdot \left( D_2 \nabla v - v\Big [(D_1-D_3)\nabla u+ (D_2-D_3)\nabla v\Big ] \right) \end{array} \right. \end{aligned}$$

It is more convenient to use the same change of variables *u*, *v* to *p*, *q* as in (5): $p=u+v$, $q=D_1 u+D_2 v.$ For these functions we obtain the system of differential equations

7 $$\begin{aligned} \left\{ \begin{array}{ll} \frac{\partial p}{\partial t} =&{} \nabla \cdot \left[ \nabla q - p \nabla (q-\varepsilon p) \right] \\ \frac{\partial q}{\partial t} =&{} \nabla \cdot \left[ -D_1 D_2 \nabla p+(D_1+D_2)\nabla q - q\nabla (q-\varepsilon p)\right] \end{array} \right. \end{aligned}$$

where $\varepsilon := D_3$. Observe that if $\varepsilon =0$ then (7) is equivalent to (6). We expand the functions *p* and *q* in the series with respect to the small parameter $\varepsilon$:

8 $$\begin{aligned} p=&p^{[0]}+\varepsilon p^{[1]}+\varepsilon ^2 p^{[2]}+\cdots =\sum _{l=0}^{\infty }\varepsilon ^l p^{[l]},\nonumber \\ q=&q^{[0]}+\varepsilon q^{[1]}+\varepsilon ^2 q^{[2]}+\cdots =\sum _{l=0}^{\infty }\varepsilon ^l q^{[l]}. \end{aligned}$$

Once we substitute these expansions to the system (7) and compare the coefficients we get the recurrence relation

9 $$\begin{aligned} \left\{ \begin{array}{ll} \frac{\partial p^{[k]}}{\partial t}+{}\nabla \cdot \Big (p^{[k]}\nabla q^{[0]}\Big )&=-\sum_{l=1}^{k-1} \nabla \cdot \Big (p^{[l]} \nabla q^{[k-l]}\Big )\\ & \nonumber \quad+ \sum _{l=0}^{k-1} \nabla \cdot \Big (p^{[l]} \nabla p^{[k-l-1]}\Big )\\ \frac{\partial q^{[k]}}{\partial t}-{}\Big (D_1+D_2-q^{[0]}\Big ) \nabla ^2 q^{[k]}+2\nabla q^{[0]}\cdot \nabla q^{[k]}&=-D_1 D_2 \nabla ^2 p^{[k]}\\ &{} \quad -\sum _{l=1}^{k-1} \nabla \cdot \Big (q^{[l]} \nabla q^{[k-l]}\Big ) \\ & \nonumber \quad+ \sum _{l=0}^{k-1} \nabla \cdot \Big (q^{[l]} \nabla p^{[k-l-1]}\Big ) \end{array} \right. \end{aligned}$$

for $k\ge 0$. We represent the initial conditions for the unknown functions $p^{[k]}$ and $q^{[k]}$ as follows:

10 $$\begin{aligned} p^{[0]}(0,x)&=\frac{u_0(x)+v_0(x)}{1-w_0(x)}=p_0(x)\sum _{l=0}^{\infty }w_0^l(x),\nonumber \\ q^{[0]}(0,x)&=\frac{D_1 u_0(x)+D_2 v_0(x)}{1-w_0(x)}=q_0(x)\sum _{l=0}^{\infty }w_0^l(x), \end{aligned}$$

where $p_0=u_0+v_0$, $q_0=D_1u_0+D_2v_0$. It is easy to see that $p^{[0]}(0,x)=\frac{1-w_0(x)}{1-w_0(x)}\equiv 1$. If we compare the right-hand sides of (10) and (8), we obtain the initial conditions for the unknown coefficients $p^{[k]}, q^{[k]}$ for $k\ge 1$:

$$\begin{aligned} p^{[k]}(0,x)=-p_0(x)\frac{w_0^k(x)}{\varepsilon ^k},\;\;\; q^{[k]}(0,x)=-q_0(x)\frac{w_0^k(x)}{\varepsilon ^k} \end{aligned}$$

for $$x\in \bar{\Omega }$$.

Using a small parameter expansion of functions *p* and *q* we replace the original system of equations (7) by a separate systems of equations (9) for each pair of functions $(p^{[k]},q^{[k]})$, $k\ge 0$. The complexity of these systems is rising with increasing values of *k*. Therefore, the expansions (8) are practically limited to several terms. According to this we are mainly interested in the asymptotic nature of this truncated expansion for $\varepsilon \rightarrow 0$, $\varepsilon \ll 1$, then the possible divergence of the whole series is in general not important.

The study of asymptotic expansions was first made by Poincaré. According to Poincaré’s definition, a series $\sum \nolimits _{n=0}^{\infty }p^{[n]}(t,x;\varepsilon )\varepsilon ^n$ is said to be asymptotic to a function $p(t,x;\varepsilon )$, i.e.

$$\begin{aligned} p(t,x;\varepsilon )\sim \sum _{n=0}^{\infty }p^{[n]}(t,x;\varepsilon )\varepsilon ^n, \end{aligned}$$

iff for every $N\ge 0$ and $\varepsilon \rightarrow 0$, $\varepsilon \ll 1$, we have

$$\begin{aligned} p(t,x;\varepsilon )-\sum _{n=0}^{N}p^{[n]}(t,x;\varepsilon )\varepsilon ^n=o(\varepsilon ^N). \end{aligned}$$

This definition is equivalent to the following conditions

$$\begin{aligned} \lim _{\varepsilon \rightarrow 0}\frac{p(t,x;\varepsilon )}{\sum _{k=0}^{\infty }p^{[k]}(t,x;\varepsilon )\varepsilon ^k}=1\quad \text {or} \quad \lim _{\varepsilon \rightarrow 0}\frac{p(t,x;\varepsilon )-\sum _{k=0}^{N}p^{[k]}(t,x;\varepsilon )\varepsilon ^k}{\varepsilon ^N}=0. \end{aligned}$$

Taking the first limit under consideration, we have

$$\begin{aligned} \lim _{\varepsilon \rightarrow 0}\frac{p(t,x;\varepsilon )}{\sum _{k=0}^{\infty }p^{[k]}(t,x;\varepsilon )\varepsilon ^k}= \frac{p(t,x;0)}{p^{[0]}(t,x;0)}. \end{aligned}$$

Since $D_3=\varepsilon \rightarrow 0$, the functions *p*(*t*, *x*; 0), $p^{[0]}(t,x;0)$ become solutions of the same initial boundary value problem which leads to the conclusion that the limit is equal to 1 for every *N*. The same result we obtain for the expansion of $q(t,x;\varepsilon )$. Therefore, the expansions (8) are asymptotic if only $\varepsilon \ll 1$. Even if the series do not converge, the asymptotic expansions give a good approximation of solutions if we take under consideration only few terms of these expansions with a very small value of $\varepsilon$ [11, 20]. This conclusion is confirmed by our numerical analysis in one dimensional case presented in Example 1. It turns out that our method is very effective for multidimensional couples, even in the case of complicated geometries.

## Numerical Analysis

This section is devoted to a numerical analysis of solutions to the problem (9), (10), obtained by the small parameter expansion method. We construct a conservative difference scheme and present the numerical behavior of ternary solid solutions based on this method.

### Finite Difference Method

We discretize the system of partial differential equations (9). Consider a uniform partition of $[0,T]\times \bar{\Omega }$, where $\bar{\Omega }=[-L,L]=[-L_1,L_1]\times \cdots \times [-L_n,L_n]$. Let $K\in \mathbb {R},$ $N_1,\ldots ,N_n$ are positive integers and $N=(N_1,\ldots ,N_n).$ Let $(h_0,h)$, $h=(h_1,\ldots ,h_n)$, stand for steps of a regular mesh on $[0,T]\times \bar{\Omega }$, such that $h_0=T/K$ and $h = L / N$. For $m = (m_1, \ldots , m_n) \in \mathbb {Z}^n$ we put $x^{(m)} = ( m_1 h_1, \ldots , m_n h_n)$ and

$$\begin{aligned} \mathbb {R}_h^n = \{\, x^{(m)}:\; m \in \mathbb {Z}^n\, \},\; \bar{\Omega }_h = \bar{\Omega }\cap \mathbb {R}_h^n. \end{aligned}$$

We convey that $(p^{[k]}_h,q^{[k]}_h):=\Big ((p^{[k]})_h^{(i,m)},(q^{[k]})_h^{(i,m)}\Big )$ will represent an approximation to the exact value of the functions $(p^{[k]},q^{[k]})$ at the point $(t _i,x_m)$ for $i \in \{0 , 1 , \ldots , K\}$ and $m \in \{-N , -N+1 , \ldots ,\, N\}$.

Let $e_j=(0,\ldots ,1,\ldots 0)\in \mathbb {R}^n$, $1\le j\le n$, where 1 is the *j*th coordinate. We employ standard and linear difference operators $\delta _t$, $\delta ^{\pm }=(\delta _{1}^{\pm },\ldots ,\delta _{n}^{\pm })$, $\delta ^{(2)}=[\delta _{rj}]_{rj=1,\ldots ,n}$:

$$\begin{aligned}&\delta _t z = \frac{z^{(i+1,m)} -z^{(i,m)}}{h_0},\\&\delta _j^+z = \frac{z^{(i,m+e_j)} -z^{(i,m)}}{h_j},\quad \delta _j^-z = \frac{z^{(i,m)} -z^{(i,m-e_j)}}{h_j},\\&\delta _{jj}^{(2)}z = \frac{z^{(i,m+e_j)} -2 z^{(i,m)}+z^{(i,m-e_j)}}{h_j^2}=\delta _j^+\delta _j^-z,\;\;\; 1\le j\le n. \end{aligned}$$

Denote by $\nabla _h$ the discrete equivalent of the gradient operator $\nabla$. Let $\nabla _h^{2}=\sum _{j=1}^n\delta _{jj}^{(2)}$ then we define

$$\begin{aligned} \nabla _h^{\pm }\cdot \Big (y\nabla _h^{\mp }z\Big )&=\sum _{j=1}^n\frac{1}{2}\Big (\delta _j^{+}y\delta _j^{-}z+\delta _j^-y\delta _j^+z\Big )+y\nabla _h^2z,\\ \nabla _h^{\pm }\cdot \Big (y\nabla _h^{\pm }z\Big )&=\sum _{j=1}^n\frac{1}{2}\Big (\delta _j^{+}y\delta _j^{+}z+\delta _j^-y\delta _j^-z\Big )+y\nabla _h^2z. \end{aligned}$$

With these conventions, the finite-difference scheme will be given by the following discrete system

11 $$\begin{aligned} \left\{ \begin{array}{ll} \delta _t p_h^{[k]}&{}=-\nabla _h^{\pm }\cdot \Big (p_h^{[k]}\nabla _h^{\mp } q_h^{[0]}\Big )-\sum _{l=1}^{k-1} \nabla _h^{\pm }\cdot \Big (p_h^{[l]} \nabla _h^{\mp } q_h^{[k-l]}\Big )\\ &{} \quad + \sum _{l=0}^{k-1} \nabla _h^{\pm }\cdot \Big (p_h^{[l]} \nabla _h^{\pm } p_h^{[k-l-1]}\Big )\\ \delta _t q_h^{[k]}&{}=-D_1 D_2 \nabla _h^{2}p_h^{[k]}+(D_1+D_2) \nabla _h^{2}q_h^{[k]}-\sum _{l=0}^k \nabla _h^{\pm }\cdot \Big (q_h^{[l]} \nabla _h^{\mp } q_h^{[k-l]}\Big ) \\ &{} \quad + \sum _{l=0}^{k-1} \nabla _h^{\pm }\cdot \Big (q_h^{[l]} \nabla _h^{\pm } p_h^{[k-l-1]}\Big ) \end{array} \right. \end{aligned}$$

with the discrete initial conditions

12 $$\begin{aligned} (p_h^{[k]})^{(0,m)}= p^{[k]}(0,x^{(m)}), \;\;\; (q_h^{[k]})^{(0,m)}= q^{[k]}(0,x^{(m)}). \end{aligned}$$

The discrete Neumann boundary conditions have the form

13 $$\begin{aligned} &(p_h^{[k]})^{(i,N+1)}= (p_h^{[k]})^{(i,N-1)}, (q_h^{[k]})^{(i,N+1)}= (q_h^{[k]})^{(i,N-1)},\nonumber \\ & (p_h^{[k]})^{(i,-N-1)}= (p_h^{[k]})^{(i,-N+1)}, (q_h^{[k]})^{(i,-N-1)}= (q_h^{[k]})^{(i,-N+1)}, \end{aligned}$$

for $$i\in \{0,\ldots , K\}$$.

### Numerical Simulations

In this section we explore the capability of the constructed method (11)–(13) to provide good approximations to the exact solution of the PDE problem (1)–(3). We present some examples of tracer and up-hill diffusion in $\mathbb {R}^1$ and tracer diffusion in $\mathbb {R}^2$. Examples in one-dimensional case are similar to those presented in [16]. Therefore, one can compare the results of the method of lines obtained in [16] with the results obtained by our numerical scheme. We also provide more complicated two-dimensional cases which are absent from the literature.

#### *Example 1*

(Tracer at interface) Let $n=1$. In the numerical analysis we take $L=1$ and the diffusion parameters $D_1=0.18, D_2=0.08, D_3=\varepsilon$. The initial distribution $u_0$ is a small perturbation of the Heaviside function and $v_0$ is a symmetry of $u_0$ about the ordinate axis. The solution *w* is initiated by a smoothed small peak

14 $$\begin{aligned} u_0(x)=&\Psi (100x)-\Phi (100x)+\mathbf {1}_{(0.01,1]}(x)\nonumber \\ v_0(x)=&u_0(-x),\quad w_0(x)=2\Phi (100x), \end{aligned}$$

where $\mathbf {1}_{A}$ denotes the characteristic function of a set *A*, and

$$\begin{aligned} \Psi (x)=&\frac{-x^3+3x+2}{4}\mathbf {1}_{[-1,1]}(x),\\ \Phi (x)=&\frac{(x^2-1)^2}{40}\mathbf {1}_{[-1,1]}(x) \end{aligned}$$

for $x\in [-1,1]$. We analyze the results for the PDE system (1) with the initial distributions given by (14) with respect to the small parameter $\varepsilon$. Illustrative simulations are provided with: $h_0=2\cdot 10^{-5}$ and $h=2\cdot 10^{-2}$. Based on formula (5) we can derive

15 $$\begin{aligned} u^{[i]}=\frac{D_2p^{[i]}-q^{[i]}}{D_2-D_1},\;\;\; v^{[i]}=\frac{q^{[i]}-D_1p^{[i]}}{D_2-D_1},\;\;\; w^{[i]}=1-u^{[i]}-v^{[i]}, \;\;\; i=0,\ldots ,k. \end{aligned}$$

**Fig. 3:**
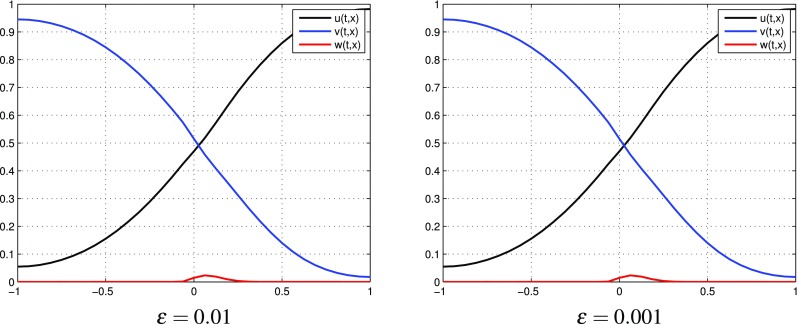
(Tracer at interface) the case $k=1$: partial sums $u^{[0]}+\varepsilon u^{[1]},\, v^{[0]}+\varepsilon v^{[1]}, w^{[0]}+\varepsilon w^{[1]}$ for different values of $\varepsilon$ at time $t=0.8$
- $k=2$, three terms in each expansions: $p=p^{[0]}+\varepsilon p^{[1]}+\varepsilon ^2 p^{[2]}$, $q=q^{[0]}+\varepsilon q^{[1]}+\varepsilon ^2 q^{[2]}$. Figure 4 presents the behavior of $u^{[2]}, v^{[2]}$, $w^{[2]}$ computed by (15). Numerical results of partial sums $u^{[0]}+\varepsilon u^{[1]}+\varepsilon ^2 u^{[2]}, v^{[0]}+\varepsilon v^{[1]}+\varepsilon ^2 v^{[2]}, w^{[0]}+\varepsilon w^{[1]}+\varepsilon ^2 w^{[2]}$ are presented in Fig. 5. Similarly as in the case $k=1$, it can be observed that the oscillations of $u^{[2]}, v^{[2]}$, $w^{[2]}$ increase. Unlike the case $k=1$, the partial sums for *u*, *v*, *w* take values in [0, 1] for sufficiently small $\varepsilon$. If $\varepsilon =0.01$, the partial sums for *u*, *v* are not monotone. In Fig. 6 one can observe additional results for partial sums $u^{[0]}+\varepsilon u^{[1]}+\varepsilon ^2 u^{[2]}, v^{[0]}+\varepsilon v^{[1]}+\varepsilon ^2 v^{[2]}, w^{[0]}+\varepsilon w^{[1]}+\varepsilon ^2 w^{[2]}$ at times $t=0.1$ and $t=0.2$ with fixed $\varepsilon =0.001$. We present these results to illustrate the dynamics of approximate distributions.
  **Fig. 4:**
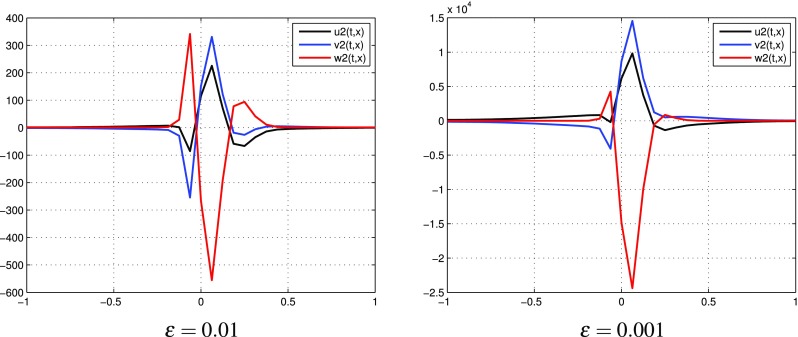
(Tracer at interface) the case $k=2$: functions $u^{[2]},\, v^{[2]}, w^{[2]}$ for different values of $\varepsilon$ at time $t=0.8$

We consider expansions (8) for $k=0,1,2$:

- $k=0$, one term in each expansions: $p=p^{[0]}$, $q=q^{[0]}$. Numerical results for the respective $u^{[0]}$, $v^{[0]}$, $w^{[0]}$ calculated by (15) are presented in Fig. 1. Note that we obtain an expected behavior of densities *u*, *v*, *w* regardless of the $\varepsilon$ value selection.
  **Fig. 1:**
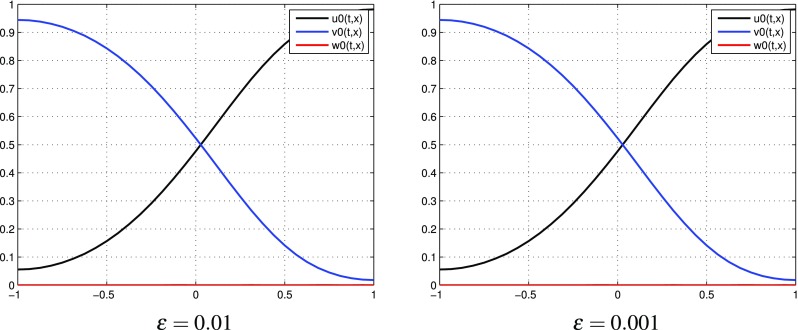
(Tracer at interface) the case $k=0$: functions $u^{[0]},\, v^{[0]}, w^{[0]}$ for different values of $\varepsilon$ at time $t=0.8$
- $k=1$, two terms in each expansions: $p=p^{[0]}+\varepsilon p^{[1]},$ $q=q^{[0]}+\varepsilon q^{[1]}$. In Fig. 2 we observe the distributions of $u^{[1]}, v^{[1]}, w^{[1]}$ computed by (15) with different values of $\varepsilon$. Figure 3 presents the behavior of partial sums $u^{[0]}+\varepsilon u^{[1]}, v^{[0]}+\varepsilon v^{[1]}, w^{[0]}+\varepsilon w^{[1]}$ with different values of $\varepsilon$. Although oscillations of $u^{[1]}, v^{[1]}, w^{[1]}$ increase with decreasing values of $\varepsilon$, we obtain reasonable approximations of *u*, *v*, *w* by means of partial sums $u^{[0]}+\varepsilon u^{[1]},$ $v^{[0]}+\varepsilon v^{[1]}, w^{[0]}+\varepsilon w^{[1]}$, respectively. Notice that these partial sums take values in the interval [0, 1] for all $\varepsilon$.
  **Fig. 2:**
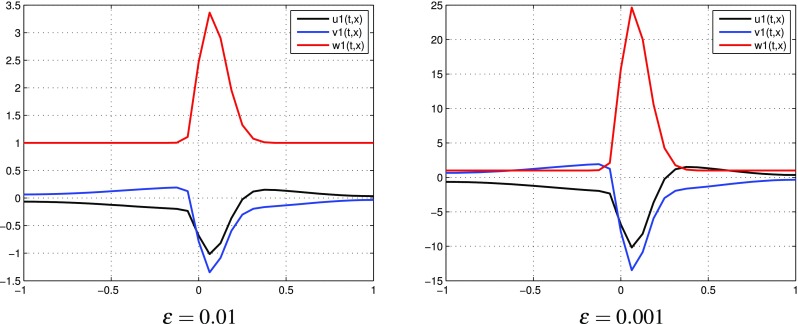
(Tracer at interface) the case $k=1$: functions $u^{[1]},\, v^{[1]}, w^{[1]}$ for different values of $\varepsilon$ at time $t=0.8$

**Fig. 5:**
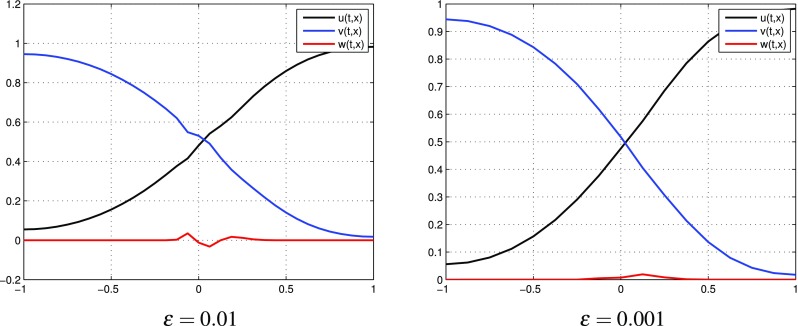
(Tracer at interface) the case $k=2$: partial sums $u^{[0]}+\varepsilon u^{[1]}+\varepsilon ^2 u^{[2]},\, v^{[0]}+\varepsilon v^{[1]}+\varepsilon ^2 v^{[2]},\, w^{[0]}+\varepsilon w^{[1]}+\varepsilon ^2 w^{[2]}$ for different values of $\varepsilon$ at time $t=0.8$

**Fig. 6:**
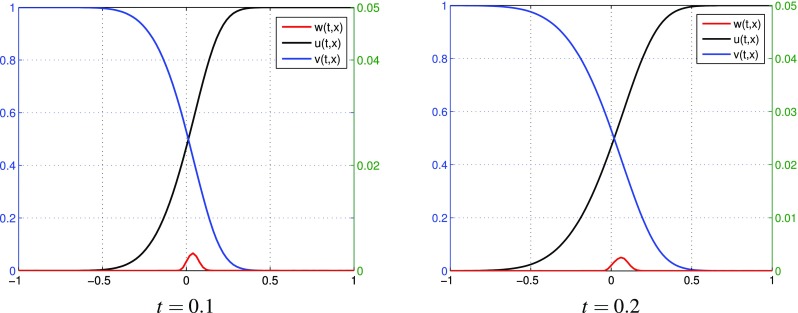
(Tracer at interface) the case $k=2$: partial sums for *u*, *v*, *w* at time $t=0.1$ and $t=0.2$

We conclude that the number of terms in the expansions (8) is closely related to the size of the parameter $\varepsilon.$ Once we use more terms in the expansions, we need to decrease the value of $\varepsilon$ to get reasonable approximation of *u*, *v*, *w* by means of respective partial sums, i.e. taking values in the interval [0, 1] and preserving monotonicity of partial sums for *u*, *v*.

#### *Example 2*

(Up-hill diffusion) We consider the one-dimensional case and we set $L=1$ similarly as in Example 1. We take $D_1=0.2, D_2=0.08, D_3=0.001=\varepsilon$. We put some monotone functions as initial distributions of substances *u*, *v* and we assume a constant initial distribution *w*:

$$\begin{aligned}&u_0(x)=\Psi (100x)+0.49\mathbf {1}_{[-0.01,0.01]}(x)+0.98\mathbf {1}_{(0.01,1]}(x),\\&v_0(x)=u_0(-x),\quad w_0(x)=0.02, \end{aligned}$$

where

$$\begin{aligned} \Psi (x)=\left( \frac{30}{176}x^5-\frac{50}{88}x^3+\frac{139}{176}x\right) \mathbf {1}_{[-1,1]}(x). \end{aligned}$$

**Fig. 7:**
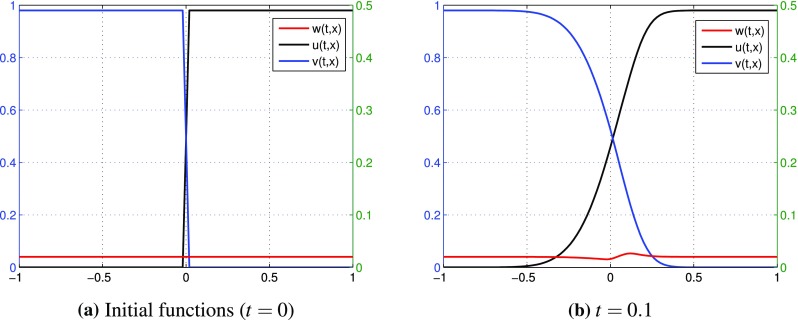
(Up-hill diffusion) **a** initial functions $u_0,\, v_0,\, w_0$, **b** partial sums $u^{[0]}+\varepsilon u^{[1]}+\varepsilon ^2 u^{[2]}, v^{[0]}+\varepsilon v^{[1]}+\varepsilon ^2 v^{[2]}, w^{[0]}+\varepsilon w^{[1]}+\varepsilon ^2 w^{[2]}$ at time $t=0.1$

Results of numerical simulations for partial sums $u^{[0]}+\varepsilon u^{[1]}+\varepsilon ^2 u^{[2]}, v^{[0]}+\varepsilon v^{[1]}+\varepsilon ^2 v^{[2]}, w^{[0]}+\varepsilon w^{[1]}+\varepsilon ^2 w^{[2]}$ computed with $h_0=2\cdot 10^{-5}, h=2\cdot 10^{-2}$ are given in Fig. 7(b) at time $t=0.1$. Figure 7a presents initial functions $u_0, v_0, w_0$.

No numerical analysis for tracer diffusion in two-dimensional case have been done so far in the literature because of the computational complexity, not to mention any efficient comparison of numerical results with real-life experiments. Due to our approach we constructed a numerical method which allows to perform computations even for multi-dimensional cases. We present two examples of numerical simulations for tracer diffusion in $\mathbb {R}^2$.

#### *Example 3*

**Fig. 8:**
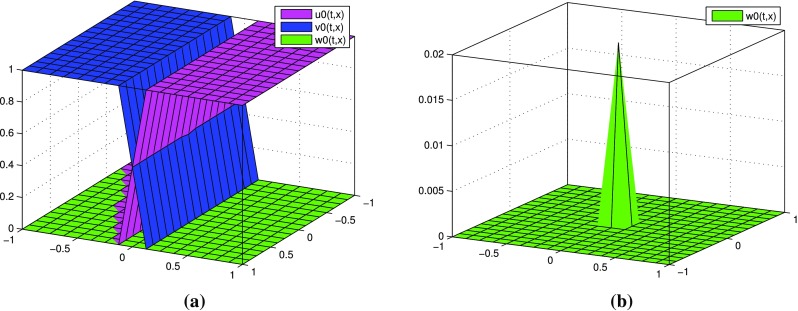
(Tracer diffusion) initial distributions: **a** $u_0, v_0, w_0$, **b** enlargement of $w_0$

(Tracer diffusion ) Consider the case $n=2$. Set $L=1$ and $D_1=0.2, D_2=0.08, D_3=0.001=\varepsilon$. Initial functions (Fig. 8) are given by

$$\begin{aligned} u_0(x,y)=&\theta (100x)-\phi (100x,100y)+\mathbf {1}_{(0.01,1]}(x),\\ v_0(x,y)=&u_0(-x,y),\quad w_0(x,y)=2\phi (100x,100y), \end{aligned}$$

where

$$\begin{aligned}&\phi (x,y)=\frac{(x^2+y^2-1)^2}{100}\mathbf {1}_{(x\in [-1,1],y\in [-1,1])}(x,y)\mathbf {1}_{x^2+y^2\le 1}(x,y),\\&\theta (x)=\frac{-x^3+3x+2}{4}\mathbf {1}_{[-1,1]}(x). \end{aligned}$$

**Fig. 9:**
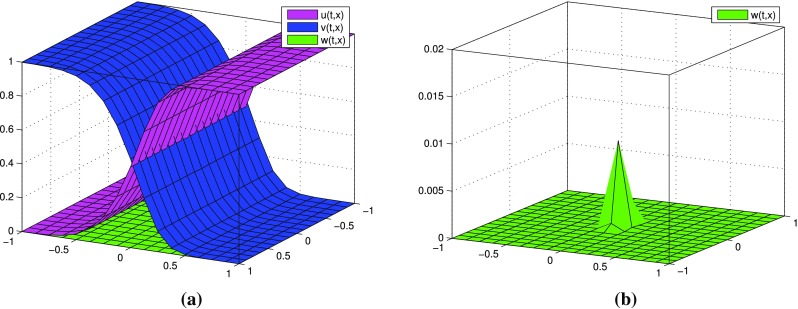
(Tracer diffusion) partial sums with $k=2$ at time $t=0.2$: **a** for *u*, *v*, *w*, **b** enlargement of *w*

In Fig. 9a we observe approximate distributions of partial sums for *u*, *v*, *w* with $k=2$ and the step sizes $h_0=2^{-15}$, $h=2^{-3}$ at time $t=0.2$. Figure 9b presents enlargement of *w*.

In the above example initial distribution of *u* and *v* is symmetrical with respect to the plane $x=0$. Now we present two-dimensional example where the symmetry of initial distribution is broken.

#### *Example 4*

**Fig. 10:**
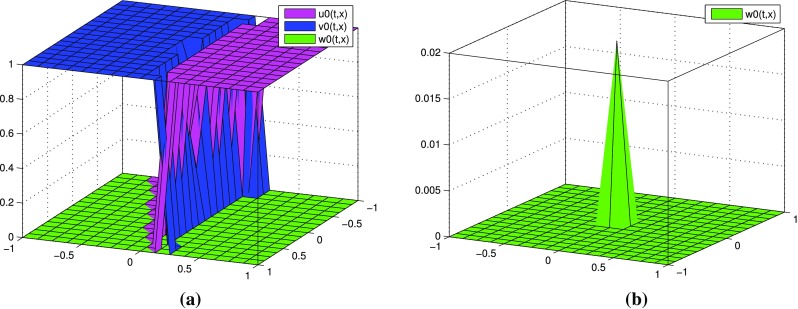
(Tracer diffusion) initial distributions: **a** $u_0, v_0, w_0$, **b** enlargement of $w_0$

**Fig. 11:**
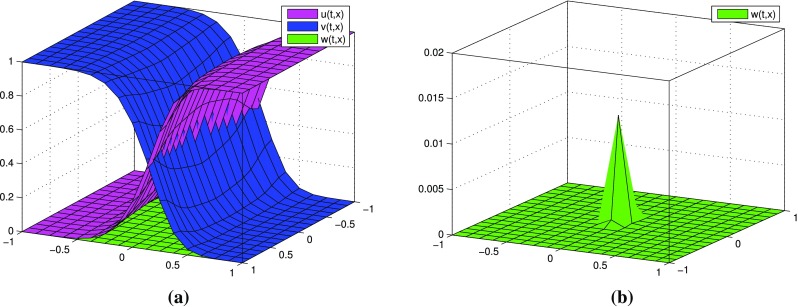
(Tracer diffusion) partial sums with $k=2$ at time $t=0.2$: **a** for *u*, *v*, *w*, **b** enlargement of *w*

(Tracer diffusion) Consider the case $n=2$. Set $L=1$ and $D_1=0.15, D_2=0.08, D_3=0.001=\varepsilon$. Initial functions (Fig. 10) are given by

$$\begin{aligned}&u_0(x,y)=\theta (100(x-0.2y^2))-\phi (100x,100y)+\mathbf {1}_{(0.01+0.2y^2,1]}(x),\\&v_0(x,y)=\theta (-100(x-0.2y^2))-\phi (100x,100y)+\mathbf {1}_{[-1,-0.01+0.2y^2)}(x),\\&w_0(x,y)=2\phi (100x,100y), \end{aligned}$$

where $\phi$ and $\theta$ are the same as in Example 3. We perform simulations with parameters: the step sizes $h_0=2^{-15},$ $h=2^{-3}$ and the length of series expansions $k=2$. In Fig. 11(a) the approximate distributions of partial sums for *u*, *v*, *w* at time $t=0.2$ are presented. Figure 11b shows enlargement of *w*.

We conduct a brief analysis of marker positions in time, see [21]. By ‘marker’ we understand a small amount of substance placed in the diffusion couple, this substance moves according to locally not-balanced diffusive fluxes. Recall that diffusive fluxes are proportional to gradients of *u*, *v*, *w*. In the above tracer diffusion examples the marker is represented by the substance *w*. Referring to Example 1 the trajectory of the marker position is described by the ODE initial value problem

16 $$\begin{aligned} \left\{ \begin{array}{ll} {z}^\prime(t) = &{} \nu ^{D}(t,z(t))=D_1u_x+D_2v_x+D_3w_x,\\ z(0) = &{} 0,\end{array}\right. \end{aligned}$$

where $\nu ^{D}$ is the drift velocity. Since the diffusion coefficient $D_3$ is small, it can be omitted,

$$\begin{aligned} \nu ^{D}(t,z(t))\approx q_x(t,z(t))=D_1u_x+D_2v_x, \end{aligned}$$

then the solution of the Cauchy problem (16) can be approximated by the solution of the following ODE problem

17 $$\begin{aligned} \left\{ \begin{array}{ll} z'(t) = &{} q_x(t,z(t))=D_1u_x+D_2v_x,\\ z(0) = &{} 0. \end{array} \right. \end{aligned}$$

**Fig. 12:**
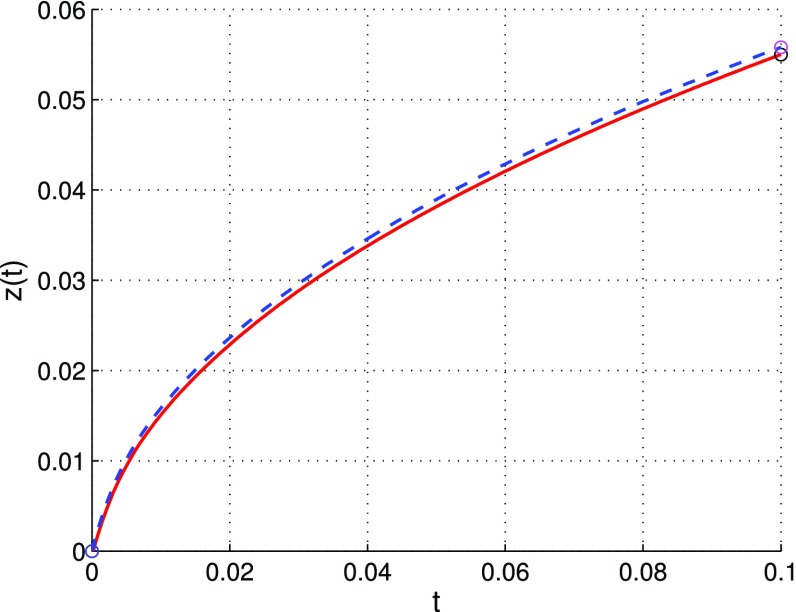
The trajectory of marker position—approximate solutions of problems (16) (*solid line*) and (17) (*dashed line*) for $h_0=2\cdot 10^{-5},\, h=2\cdot 10^{-2}$

To solve the problems (16) and (17) numerically, we first use a polynomial fitting for discrete functions *u*, *v*, *w* with respect to spatial variable. Then approximate solutions to the above ODE problems are computed by a second-order Runge–Kutta method. The results of computations with step sizes: $h_0=2 \times 10^{-5}, h=2 \times 10^{-2}$ are illustrated in Fig. 12. A mesh refinement leads to better accuracy, i.e. the solution to problem (17) gives a very good approximation of the solution to problem (16). An analogous discussion can be conducted for Examples 3 and 4.

## Conclusions and Remarks

Before we close this paper with concluding remarks we would like to mention other applications of asymptotic expansion methods, in addition to those given in the introduction, to point out the importance of these techniques in a different branches of knowledge. Some applications can be observed in the fluid and solid mechanics theory. In [22] the asymptotic expansion method was used as a tool to derive a new two-dimensional shallow water model from the time-averaged non-dimensional Navier–Stokes equations. Asymptotic simplification procedures for linear and nonlinear wave propagation problems that contain large parameters are examined in [20]. A divergent asymptotic series found applications also in the boundary layer theory [23] and in the nonlinear wave theory [24].

We use the asymptotic expansion techniques to the ternary diffusion problem. Modeling of interdiffusion phenomena is not yet unified, and various methods differ from each other in the arbitrary choice of the reference velocity, i.e. the drift or convection velocity. In computational solid mechanics and physics it is defined as being equal to the mass average velocity [13]. In gases and fluids the drift is defined based on the volume average velocity [25]. It is still common to neglect drift (convection) and assume Fickian diffusion [26]. Such simplified approach to the mass transport was used there to a three-dimensional model of interdiffused quantum dots. The above methods do not allow considering all interdiffusion effects [26]. The use of widely accepted Onsager method [27, 28] is narrow by very limited data on transport coefficients. In material sciences and chemistry the drift definition bases on the Darken method [29, 30]. The Darken’s basic postulate is that the reference velocity in multicomponent systems coincides with the drift velocity [30]. The drift in solids is often the vacancy flux generated during an interdiffusion process caused by the difference in the diffusion coefficients. The compatibility of Darken and Onsager formalisms has been already proved [31]. Onsager’s and Fickian laws are known in life sciences, see [14].

In this paper we consider a strongly coupled system of diffusion equations with Neumann boundary conditions. This system is strongly coupled, highly nonlinear and possesses a parabolic type only in a very restricted subregion of the phase space, which is related to the mass conservation. This causes analytical and numerical difficulties. To overcome these difficulties we use asymptotic expansions of solutions in divergent series with respect to a small parameter $\varepsilon :=D_3$. We obtain another system of equations which is more convenient to analyze. Our examples described in the previous section are based on this method and illustrate the behavior of ternary solid solutions (alloys), in particular: up-hill diffusion in $\mathbb {R}^1$, tracer diffusion in $\mathbb {R}^1$ and tracer diffusion in a complex geometry in $\mathbb {R}^2$. We observe that solutions of the new system obtained by the expansion method with several terms, e.g. $k=1$ or $k=2$, reflect an expected behavior of solutions of the original diffusion problem. There are many open questions in the theory and practice of small parameter expansions:

- Whether or not it is possible to extend the small parameter method onto the case of singularities [11]? In the case of ternary diffusion problems singularities may be caused by irregularity of initial distributions *u*, *v*, *w*, e.g. Heaviside or smoothed peak type functions.
- Is it possible to apply the small parameter method to a derivation of interfaces, e.g. via Matano’s method [32]?
- If indeed replacing Neumann boundary conditions by Stefan free boundary conditions is easy to conduct?
- Is it possible to generalize our method onto the case of media with variable diffusion coefficients and multi-phase media?
- Is it possible to formulate and perform computations by means of a small parameter method within Onsager’s formalism? [33–35]
